# Senescence caused by telomerase inactivation in myeloid, mesenchymal, and endothelial cells has distinct effects on cancer progression

**DOI:** 10.18632/aging.206268

**Published:** 2025-06-05

**Authors:** Joseph Rupert, Zhanguo Gao, Yongmei Yu, Mikhail G. Kolonin

**Affiliations:** 1 The Brown Foundation Institute of Molecular Medicine for the Prevention of Human Diseases, McGovern Medical School, The University of Texas Health Sciences Center at Houston, Houston, TX 77030, USA

**Keywords:** senescence, telomerase, myeloid, mesenchymal, endothelial

## Abstract

The effects of cell senescence in individual cell populations of the tumor microenvironment (TME) on cancer progression remain unclear. Here, we investigated the effects of cell senescence caused by inactivation of the catalytic subunit of telomerase (Tert) in distinct TME components. We generated genetic *Tert* knockout (KO) mice driven by the *LysM* promoter in myeloid cells, by the *Pdgfra or Pdgfrb* promoter in mesenchymal cells, and by the *Tie2e* promoter in endothelial cells. We compared the effect of the *Tert* KOs in syngeneic models of orthotopically grafted E0771 breast adenocarcinoma, RM1 prostate adenocarcinoma, and KPC pancreatic adenocarcinoma. Tumors in *LysM-Tert*-KO, *Pdgfra-Tert*-KO, and *Pdgfrb-Tert*-KO mice displayed increased myofibrogenesis and desmoplasia. Tumors in *Tie2e-Tert*-KO mice displayed endothelial abnormality and the strongest reduction in tumor vascularization. This was linked with increased HIF1a protein nuclear localization, indicative of hypoxia, and the highest protein expression of the glycolytic marker GLUT1 in cancer cells. KPC tumors displayed reduced epithelial cytokeratin-19 protein expression and reduced tumor growth in all *Tert* KO models. However, liver metastases of KPC cells were only observed for *Tie2e-Tert*-KO mice. We conclude that senescence of distinct cells in the TME has different effects on cancer progression and that endothelial cell function preservation is important in metastasis suppression.

## INTRODUCTION

During aging, tissues progressively accumulate senescent cells [1, 2]. Cell senescence is the state of irreversible proliferation arrest linked with the senescence-associated secretory phenotype (SASP) and increased expression of senescence-associated beta-galactosidase (SA-βgal) and DNA damage response genes [3, 4]. Accumulation of senescent cells contributes to changes leading to metabolic dysfunction [5, 6] and underlies the development of cardiovascular diseases [7]. Adenocarcinoma development and its progression to lethal metastatic stage is also linked with aging at the tissue and cell level [8]. Most animal studies on cancer, performed in young mice, are likely to miss important nuances related to the aging tumor microenvironment (TME). There is a lack of consensus on the role of cell senescence in the TME on cancer progression [9, 10]. This could be due to senescence in distinct TME components, namely hematopoietic, mesenchymal, and endothelial cells having different effects. To address this, cancer needs to be analyzed in the context of senescence induction in individual lineages.

A key gene protecting cells from senescence is the catalytic subunit of telomerase reverse transcriptase (*TERT*), which codes for the enzyme subunit that, in complex with TERC, lengthens telomeres, prevents chromosome attrition, and supports genome integrity and mitochondria through its non-canonical functions [7, 11]. There are substantial differences in telomerase biology in mice and humans. Human telomerase is active in stem cells but is inactive in most somatic cells, which permits telomere erosion and cell aging. Replicative senescence is delayed in mice because they have relatively long telomeres and continue to express *TERT* in most somatic cells [12, 13], hence providing mice as a convenient model to study TERT function. To “humanize” the senescence onset in mice we have established mouse models of *Tert* knockout (KO) in specific tissues [14–16]. Mice with *Tert* deletion in mesenchymal stromal cells (MSC) of either *Pdgfra*+ or *Pdfrb*+ lineages were found to undergo premature telomere attrition and replicative senescence in adipocyte progenitor cells when fed a high-calorie diet. As a result, *Pdgfra*-*Tert*-KO and *Pdgfrb*-*Tert*-KO mice develop white adipose tissue (WAT) hypertrophy and pathology linked with metabolic dysfunction [16]. In contrast, the mouse endothelial cell (EC)-specific *Tert* KO model (*Tie2e*-*Tert*-KO) displays ubiquitous vasculature leakiness, tissue hypoxia, and WAT hypotrophy [14, 15]. Importantly, features of premature aging in the brain and skeletal muscle of *Tie2e*-*Tert*-KO mice develop without telomere shortening or EC depletion due to the loss of non-canonical TERT function in supporting mitochondrial biogenesis and oxidative metabolism [14].

Cancer-associated fibroblasts (CAFs), a heterogeneous and plastic population of non-malignant mesenchymal cells in the TME [17], clearly undergo senescence and have been studied the most to date. Senescent fibroblasts have been shown to promote both tumorigenesis and epithelial cell proliferation [18]. Senescent CAFs have been shown to confer chemotherapy resistance [19]. Recently, it has been shown that senescent CAFs can promote tumor growth due to their immunosuppressive function [8]. However, in other studies, senescent stromal cells have been found to suppress tumor growth [9, 10]. The senescence of other cell populations has been investigated much less thoroughly.

To investigate the effect of cell senescence in distinct types of cells in the tumor microenvironment we used lineage-specific *Tert KO* mouse models as hosts for orthotopic breast, prostate, and pancreatic cancer allografts. Myeloid cells are the main hematopoietic components of the tumor stroma [20]. To address the role of myeloid cell senescence, we created a new mouse model in which *LysM-cre* is used to knock out *Tert* (*LysM-Tert*-KO) in monocytes, macrophages, and granulocytes. We show that *Tert* knockouts in myeloid, mesenchymal, and endothelial lineages have different effects on primary tumor growth and metastasis development.

## RESULTS

### *Tert* KO in myeloid cells suppresses the growth of breast, prostate, and pancreatic tumors

**Figure 1:**
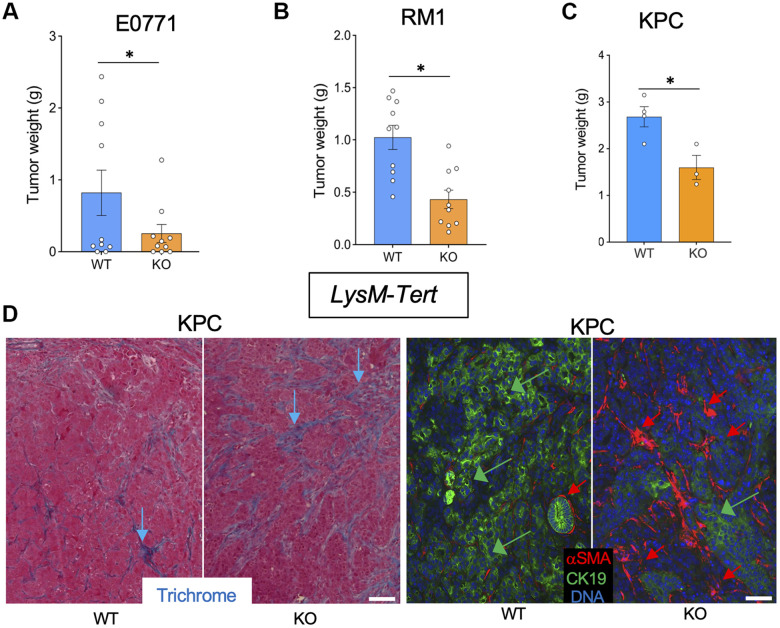
***Tert* KO in myeloid cells suppresses the growth of breast, prostate, and pancreatic tumors.** Tissues resected from *LysM-Cre; Tert^fl/fl^* (KO) and *LysM-Cre; Tert^+/+^* (WT) littermates were analyzed once one of the groups developed a critical tumor burden. (**A**) E0771 tumor weight upon resection. (**B**) RM1 tumor weight upon resection. (**C**) KPC tumor weight upon resection. (**D**) Representative paraffin sections of KPC tumors stained with Trichrome (left) or subjected to CK19 and α-SMA IF (right) revealing epithelial cells (green arrows) and myofibroblasts (red arrows). Scale bar: 100 μm. Shown are mean +/- SEM, *P<0.05, Student’s t-test.

*LysM*-*Tert*-KO and wild-type (WT) Cre+ Tert^+/+^ littermate mice were used to investigate the effect of myeloid cell senescence on cancer. E0771 tumors grown in 4 out of 8 *Tert-*WT littermates were dramatically larger than others, however H&E staining revealed increased hemorrhaging of tumors (Supplementary Figures 1A, 2A), highlighting the variability of this model. In contrast, all *LysM*-*Tert*-KO mice grafted with breast cancer E0771 cells had small tumors at the terminal time point (Figure 1A and Supplementary Figure 1A). Most *LysM*-*Tert*-KO mice grafted with prostate cancer Ras+Myc-induced (RM1) cells had smaller tumors than *Tert-*WT littermates at the terminal time point (Figure 1B and Supplementary Figure 1A). As a less hemorrhagic model, we utilized the Kras^LSL-G12D^;p53^LoxP^;Pdx1-CreER (KPC) orthotopic model of pancreatic ductal adenocarcinoma (PDAC) reported to result in spontaneous liver metastasis [21]. The majority of *LysM*-*Tert*-KO mice had smaller KPC tumors than their *Tert-*WT littermates (Figure 1C). KPC tumors from *LysM*-*Tert*-KO mice displayed more ECM deposition consistent with an increased presence of myofibroblasts expressing alpha smooth muscle actin (αSMA) (Figure 1D). Cancer cells in *LysM*-*Tert*-KO mice also tended to have a reduced expression of the epithelial marker cytokeratin 19 (CK19) suggesting their dedifferentiation (Figure 1D). Combined, these results indicate that *Tert*-KO in myeloid cells suppresses tumor growth but increases attributes of cancer aggressiveness.

### *Tert* KO in mesenchymal stromal cells suppresses the growth of pancreatic tumors

**Figure 2:**
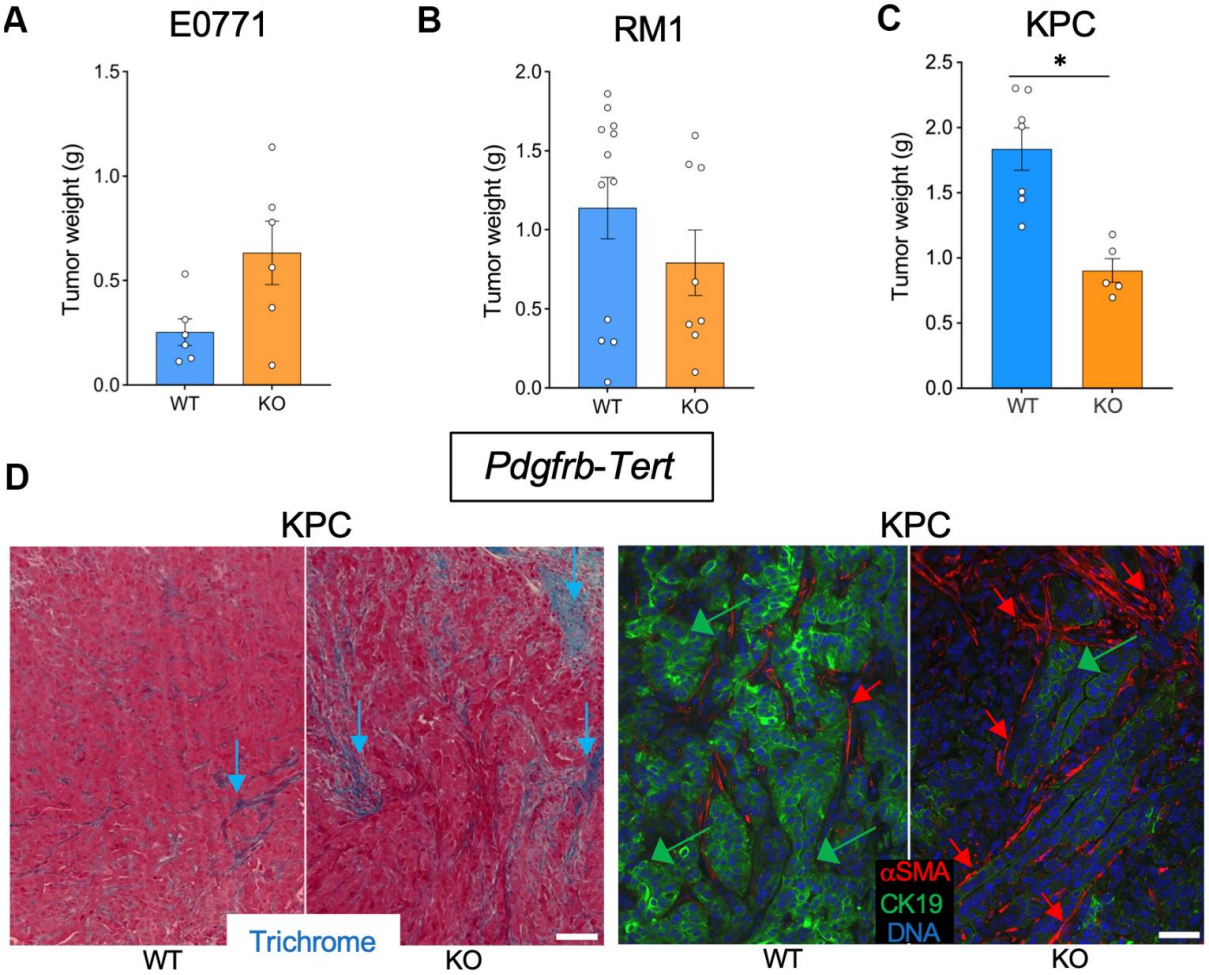
***Tert* KO in mesenchymal cells suppresses the growth of pancreatic tumors.** Tissues resected from *Pdgfrb-Cre; TERT^fl/fl^* (KO) and *Pdgfrb-Cre; Tert^+/+^* (WT) littermates were analyzed once one of the groups developed critical tumor burden. (**A**) E0771 tumor weight upon resection. (**B**) RM1 tumor weight upon resection. (**C**) KPC tumor weight upon resection. (**D**) Representative paraffin sections of KPC tumors stained with Trichrome (left) or subjected to CK19 and α-SMA IF (right) revealing epithelial cells (green arrows) and myofibroblasts (red arrows). Scale bar: 100 μm. Shown are mean +/- SEM, *P<0.05, Student’s t-test.

To investigate the effect of mesenchymal stromal cells (MSC) senescence on cancer, we used *Pdgfra*-*Tert*-KO and *Pdgfrb*-*Tert*-KO mice. Among 10 *Pdgfra*-*Tert*-KO mice grafted with breast cancer E0771 cells, only two mice grew tumors, which were significantly smaller than E0771 tumors grown in the majority of *Tert-*WT littermates (Supplementary Figures 1C, 3A). Additionally, tumors in *Pdgfra*-*Tert*-KO mice increased necrosis versus WT via H&E staining (Supplementary Figure 2C). In contrast, all *Pdgfrb*-*Tert*-KO mice grafted with E0771 cells grew tumors, some of which were larger than tumors grown in the majority of *Tert-*WT littermates (Figure 2A and Supplementary Figure 1B). However, upon fixation and sectioning, the larger tumors in *Pdgfrb*-*Tert*-KO mice were found to be to a large extent necrotic and highly hemorrhagic, with the latter accounting for their seemingly large size (Supplementary Figure 2B). *Pdgfra*-*Tert*-KO and *Pdgfrb*-*Tert*-KO mice grafted with prostate cancer RM1 cells tended to have smaller tumors at the terminal time point than *Tert-*WT littermates (Figure 2B and Supplementary Figures 1B, 1C, 3B). In KPC tumor-bearing mice, *Pdgfra*-*Tert*-KO and *Pdgfrb*-*Tert*-KO mice had significantly smaller tumors than their *Tert-*WT littermates at euthanasia (Figure 2C and Supplementary Figure 3C). Tumors from *Pdgfrb*-*Tert*-KO mice displayed more ECM deposition consistent with an increased presence of αSMA+ myofibroblasts (Figure 2D). KPC cancer cells in *Pdgfrb*-*Tert*-KO mice also tended to have a reduced expression of the epithelial marker CK19 indicating their dedifferentiation (Figure 2D). Similar changes were observed in *Pdgfra*-*Tert*-KO mice (Supplementary Figure 3D). Combined, these results indicate that *Tert*-KO in stromal cells suppresses tumor growth but increases attributes of cancer aggressiveness.

### *Tert* KO in endothelial cells suppresses the growth of breast, prostate, and pancreatic tumors

**Figure 3:**
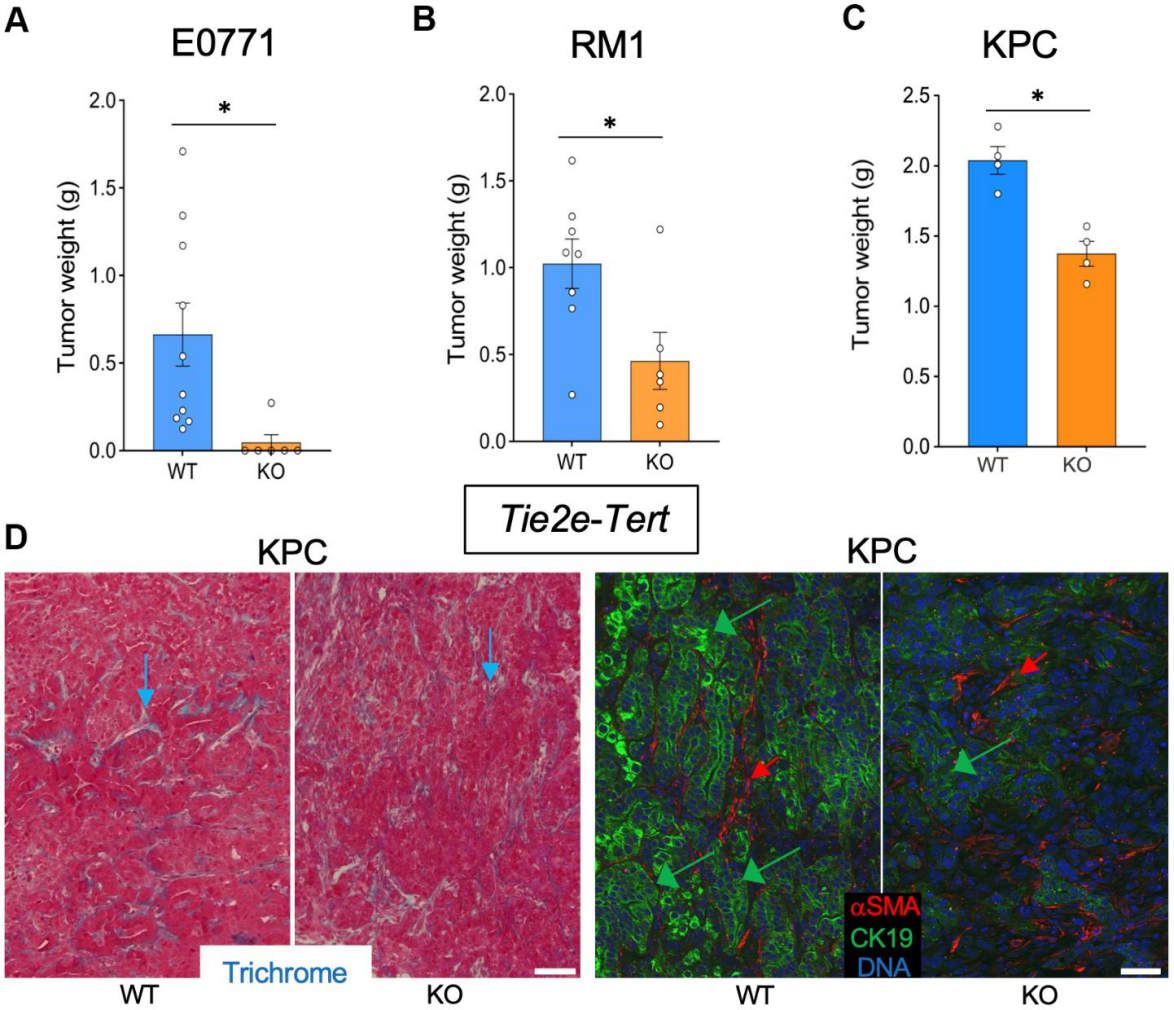
***Tert* KO in endothelial cells suppresses the growth of pancreatic tumors.** Tissues resected from *Tie2e-Cre; Tert^fl/fl^* (KO) and *Tie2e-Cre; Tert^+/+^* (WT) littermates were analyzed once one of the groups developed a critical tumor burden. (**A**) E0771 tumor weight upon resection. (**B**) RM1 tumor weight upon resection. (**C**) KPC tumor size weight upon resection. (**D**) Representative paraffin sections of KPC tumors stained with Trichrome (left) or subjected to CK19 and α-SMA IF (right) revealing epithelial cells (green arrows) and myofibroblasts (red arrows). Scale bar: 100 μm. Shown are mean +/- SEM, *P<0.05, Student’s t-test.

To investigate the effect of EC senescence on cancer, we used *Tie2e*-*Tert*-KO mice. Among six *Tie2e*-*Tert*-KO mice grafted with breast cancer E0771 cells, only one mouse grew a tumor, which was significantly smaller than E0771 tumors grown in the majority of *Tert-*WT littermates (Figure 3A and Supplementary Figure 1D). Additionally, the single E0771 tumor from the *Tie2e*-*Tert*-KO mouse had increased necrosis and hemorrhaging compared to WT via H&E staining (Supplementary Figure 2D). In contrast, all *Tert-*WT littermates had E0771 tumors variable in size. Similarly, *Tie2e*-*Tert*-KO mice grafted with prostate cancer RM1 cells had significantly smaller tumors at the terminal time point when *Tert-*WT littermates reached the critical size of 1 cm^3^ (Figure 3B and Supplementary Figure 1D). In the KPC orthotopic model, at euthanasia, *Tie2e*-*Tert*-KO mice also had significantly smaller tumors than their *Tert-*WT littermates (Figure 3C). KPC tumors from *Tie2e*-*Tert*-KO mice did not display more ECM deposition or an increase in the presence of αSMA+ myofibroblasts (Figure 3D). Cancer cells in *Tie2e*-*Tert*-KO mice tended to have a markedly reduced expression of the epithelial marker CK19 indicating their dedifferentiation (Figure 3D). These results indicate that *Tert*-KO in EC suppresses tumor growth but increases attributes of cancer aggressiveness without a notable effect on ECM.

### *Tert* KO in endothelial cells compromises the vasculature and promotes pancreatic cancer metastasis

**Figure 4:**
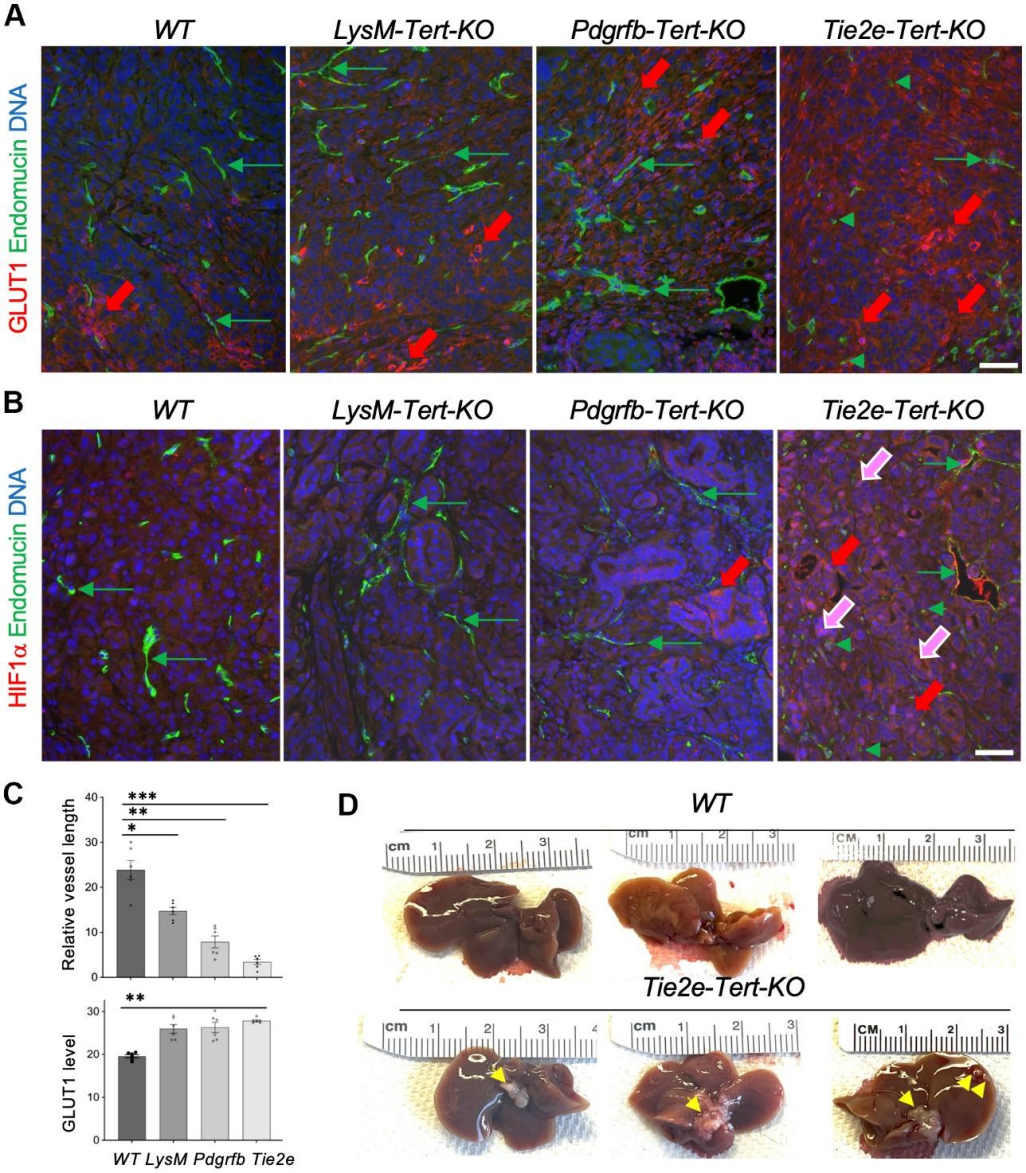
***Tert* KO in EC promotes pancreatic cancer metastasis linked with vasculature dysfunction and hypoxia.** (**A**) Paraffin sections of representative KPC tumors from EC-*Tert*-KO and WT littermates subjected to IF showing GLUT1 expression in cancer cells (red arrows) and endomucin+ endothelium (GFP). (**B**) Paraffin sections of representative KPC tumors from indicated *Tert*-KO and WT littermates subjected to IF showing HIF1α expression in cancer cells (red arrows) and nuclear localization (purple arrows). Endomucin+ IF reveals blood vessels (green arrows) and isolated EC (green arrowheads). (**C**) Quantification of mean blood vessel length (**A**, **B**) and GLUT1 IF (**A**). Shown are mean +/- SEM, *P<0.001, **P<0.0005, ***P<0.0001, ANOVA with a post-hoc test. (**D**) Representative livers from EC-*Tert*-KO and WT littermates with pancreatic KPC tumors with liver metastases indicated (arrows). DNA is blue. Scale bar: 100 μm.

To establish the mechanism responsible for tumor growth suppression in the *Tert* KO models, we analyzed KPC tumor sections by IF. Compared to WT littermates, tumors from *LysM*-*Tert*-KO, *Pdgfrb*-*Tert*-KO, and *Tie2e*-*Tert*-KO had higher GLUT1 expression in cancer cells (Figure 4A, 4C). GLUT1 expression, indicative of glycolysis induction [14], was particularly prominent in tumor areas lacking vasculature, revealed by immunofluorescence (IF) using antibodies against an endothelial marker endomucin, especially in *Tie2e*-*Tert*-KO mice (Figure 4A). Endomucin IF revealed particularly striking vasculature abnormalities in tumors of *Tie2e*-*Tert*-KO mice, compared to WT littermates and other *Tert*-KO controls (Figure 4A, 4B). Specifically, tumors in *Tie2e*-*Tert*-KO mice had vessels that were generally smaller and less patent. Moreover, many endothelial cells were dispersed in tumors in clusters lacking a vascular lumen or in isolation and were markedly large, the latter indicative of senescence (Figure 4A, 4B). To track the cell lineage of interest, we used the *mTmG* reporter cassette crossed into the experimental mice. It enabled the identification of *Tie2e+* lineage cells based on the expression of membrane GFP (mG) among cells expressing membrane Tomato (mT) as we have previously reported [14, 16]. This lineage tracing also revealed many mG+ cells being larger than in WT controls and forming non-luminal clusters (Supplementary Figure 4). The mean length of vessels observed in sections was also most strikingly reduced in *Tie2e*-*Tert*-KO mice, although also lower in *LysM*-*Tert*-KO and *Pdgfrb*-*Tert*-KO mice compared with WT littermates (Figure 4C). Importantly, increased levels of HIF1α and its nuclear localization, a marker of hypoxia, was observed specifically in tumors of *Tie2e*-*Tert*-KO mice, but not in other *Tert*-KO models or WT littermates (Figure 4B). Concordantly, liver metastases were observed for all *Tie2e*-*Tert*-KO mice but not in WT littermates (Figure 4D). Importantly, liver metastases were not observed in *LysM*-*Tert*-KO, *Pdgfra*-*Tert*-KO, or *Pdgfrb*-*Tert*-KO mice. This indicates a high degree of hypoxia as the mechanism responsible for KPC cell dissemination in EC *Tert* KO but not in the other KO models.

## DISCUSSION

The role of senescent cells in the TME is poorly understood [9, 10]. Establishing cancer-promoting and cancer-suppressing functions of individual lineages of senescent cells is important for considering them as potential therapy targets [10]. Here, by knocking out *Tert* in myeloid, mesenchymal, and endothelial lineages, we show that primary tumor growth is affected differently by cell senescence in different cell types. We also show that different cancer types can be affected differently by senescence in the same TME population. Finally, we show for the first time that metastasis is promoted by EC senescence in pancreatic cancer.

Genetic inactivation of *Tert* as a model to induce cell senescence is based on rationale from previous studies that have revealed its telomeric and non-telomeric functions [7, 11]. In addition to maintaining mitochondrial function and bioenergetics, TERT protects cells from genotoxic stress by its poorly understood global effect on the epigenome [7]. We have reported that upon *Tert* KO, actively dividing adipose stem cells undergo premature telomere attrition and become senescent [16]. However, by using mice lacking *Tert* in EC, we showed that cell senescence phenotype and upregulation of the SASP, *p53, p16, p21,* and senescence-associated β-galactosidase arise irrespective of cell proliferation and telomere attrition [14]. That study demonstrated that *Tert*-KO EC has reduced mitochondrial content and function, which results in reduced oxidative phosphorylation and increased dependence on glycolysis. In that model, *Tert*-KO vasculature develops increased vascular permeability and organ-wide tissue hypoxia, involving HIF1α activation and a metabolic switch to dependence on glycolysis [14]. In addition to replicative senescence, stress-induced senescence and oncogene-induced senescence types have been characterized. These mechanisms are likely to contribute to senescence in individual TME cells to different extents.

Among models tested here, primary tumor growth suppression, linked with dedifferentiation of epithelial cells, was observed for *Tert*-KO in every cell lineage, with one exception for E0771 grafts. *Tert*-KO in EC and *Pdgfr*+ lineage cells, detrimental to vascular function, likely affects tumor growth by restricting blood perfusion, limiting oxygenation, and nutrient access to cancer cells. *Tert*-KO in myeloid cells leads to a shift of macrophages to the pro-inflammatory phenotype (data not shown), which activates anti-tumor immunity that likely limits tumor growth in that model. Interestingly, increased myofibroblast infiltration and desmoplasia were observed not only for *Tert*-KO in *Pdgfr*+ lineages, giving rise to mesenchymal fibroblasts, but also for the *LysM*+ lineage. This is consistent with previous reports of myeloid cells contributing to the pool of CAFs and ECM deposition [17]. Remarkably, liver metastases were observed only in *Tie2e*-*Tert*-KO mice. Vascular dysfunction and the resulting hypoxia are known to force tumor cells into aggressive epigenetic states aiding metastases [22]. The specific effect of EC *Tert-*KO promoting metastatic dissemination of PDAC cells is likely due to a particularly strong tumor hypoxia induction in this model, evident from a striking HIF1a activation and nuclear localization. This is also consistent with GLUT1 upregulation, indicative of glycolytic metabolism activation, being the most pronounced in tumors of *Tie2e*-*Tert*-KO mice. Additionally, we have performed RNA-sequencing on isolated ECs from *Tie2e-cre;TertKO* mice, which showed a 3-fold increase in expression of the Procollagen-Lysine,2-Oxoglutarate 5-Dioxygenase 2 (*Plod2*) gene versus WT mice (data not shown). *Plod2* has been strongly implicated to promote epithelial to mesenchymal transition in various human cancers and preclinical models [23]. Increased *Plod2* expression in endothelial cells within the TME of *Tie2e-cre;TertKO* mice may provide a mechanism for EMT and increased liver metastasis observed with KPC tumors.

In summary, this study shows that senescence and metabolic dysfunction resulting from telomerase inactivation in distinct cells in the tumor microenvironment have different effects on tumor growth and metastasizing of carcinomas. This is an important step toward systematic characterization of the role of TME component senescence on primary tumors and their dissemination. Therapeutic approaches to the elimination of senescent cells are being developed [1, 24]. This study suggests that the effects of cell senescence in distinct cell types are cancer type-specific and calls attention to considering the potential beneficial effects of their targeting.

## MATERIALS AND METHODS

### Mouse *Tert* KO models

Mice were housed in a barrier facility with *ad libitum* access to food and water and were maintained on a twelve-hour light/dark cycle. The approach to generate *Pdgfra*-*Tert*-KO, *Pdgfrb*-*Tert*-KO, and *Tie2e*-*Tert*-KO mice and WT littermates was previously described [15, 16]; *Cre+;Tert^fl/fl^* (KO) and *Cre+;Tert^+/+^* (WT) littermates were identified by PCR genotyping. To generate mice with *Tert* KO specifically in myeloid cells, we crossed mice expressing Cre under the control of the LysM promoter [25] with Tert^fl^ mice. *LysM-Tert*-KO mice did not display notable abnormalities.

### Mouse cancer models

KPC FC1242 cells originally isolated from the KPC genetic murine model of PDAC [26] were used as described [21, 27]. Male and female 12-month-old obese mice were anesthetized using inhaled isoflurane, subcutaneously injected with Ethiqa XR (3 mg/kg), and placed in lateral recumbency on their right side. Mice were shaved and aseptically prepped. A small incision was made into the abdomen and the pancreas and spleen retracted. Using a 28G needle and a 1 mL syringe 10^5^ KPC cells were injected into the pancreas over a period of 30 seconds. The abdominal musculature was sutured, and the skin was closed using metal wound clips. For breast and prostate tumor modeling, 1×10^5^ cancer cells were grafted with a 21-gauge needle into the mammary fat pad (E0771) or subcutaneously onto the upper flank (RM1) as described [28].

### Tissue analysis

Tumors were analyzed as described [27]. Upon resection and weight measurement, portions of tumors were fixed in 10% formalin for 72 hours for paraffin embedding and tumor cross sections (5 μm) cut for histology. Trichrome blue staining was performed as described and sections were analyzed upon antigen retrieval as described [29]. Upon blocking, primary antibodies against HIF1a (GeneTex GTX127309, 1:100), endomucin (R&D Systems AF4666, 1:100), CK19 (GeneTex GTX640155, 1:100), aSMA (Sigma A2319, 1:000), and GLUT1 (Mybiosourse MBS179154, 1:100) were applied at 4° C for 12 h. IF was performed with Donkey Alexa 488-conjugated (Invitrogen A11055, 1:200) or Cy3-conjugated (Jackson ImmunoResearch 711-166-152, 1:200) IgG. IF images were acquired with a Carl Zeiss upright Apotome Axio Imager Z1 / ZEN2 Core Imaging software. All image processing and analysis were performed using ImageJ (NIH, 2023 version 1.54g). Briefly, the images were converted to 8-bit image type. The 8-bit image was set to auto threshold in image/adjust. Vascular length density (the vessel length per unit area) was measured by using the rectangular selection tool to choose the area to analyze in plugin/vessel analysis tool. This means fluorescence intensity was measured by ImageJ software to calculate the average fluorescence intensity within a selected region of an image.

### Statistics

Microsoft Excel and Graphpad Prism were used to graph data as mean ± SEM and to calculate P-values using a homoscedastic Student’s t-test or ANOVA with a post-hoc test. *P* < 0.05 was considered significant. Experiments were repeated at least twice with similar results.

## Supplementary Material

Supplementary Figures
