# Supplementary material for: Senescence caused by telomerase inactivation in myeloid, mesenchymal, and endothelial cells has distinct effects on cancer progression
Source: Aging (Albany NY). 2025 Jun 5;17(6):1452–65. doi: 10.18632/aging.206268 (PMC12245194; doi:10.18632/aging.206268)
Supplement: Supplementary Figures [file aging-17-206268-s001.pdf]

SUPPLEMENTARY FIGURES

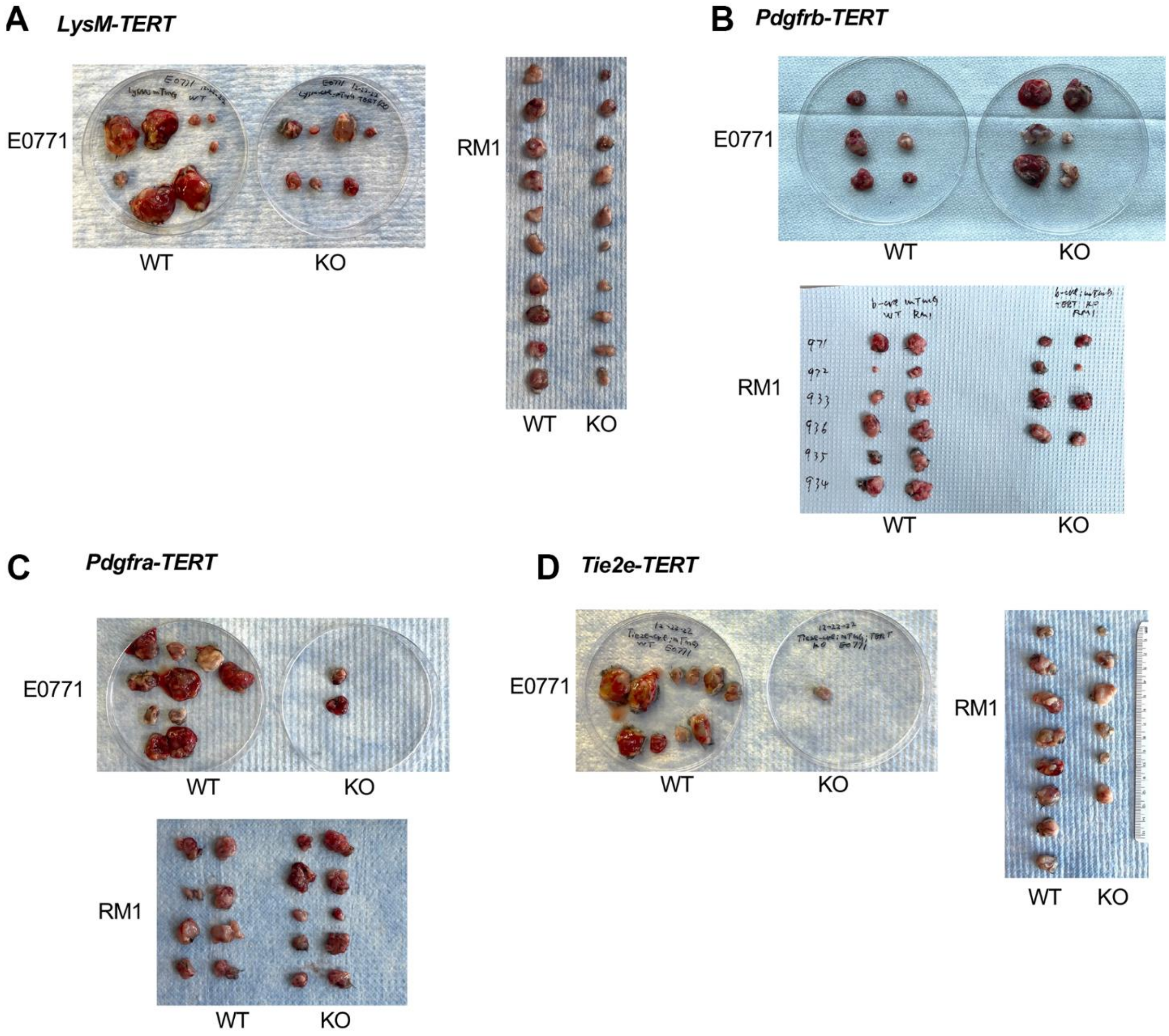

Supplementary Figure 1. Resected tumors from indicated models. (**A**) LysM-Cre; TERT$^{fl/fl}$ (KO) and LysM-Cre; TERT$^{+/+}$ (WT). (**B**) Pdgfra-Cre; TERT$^{fl/fl}$ (KO) and Pdgfra-Cre; TERT$^{+/+}$ (WT). (**C**) Pdgfrb-Cre; TERT$^{fl/fl}$ (KO) and Pdgfrb-Cre; TERT$^{+/+}$ (WT). (**D**) Tie2e-Cre; TERT$^{fl/fl}$ (KO) and Tie2e-Cre; TERT$^{+/+}$ (WT). All tumors are up to scale shown in D.

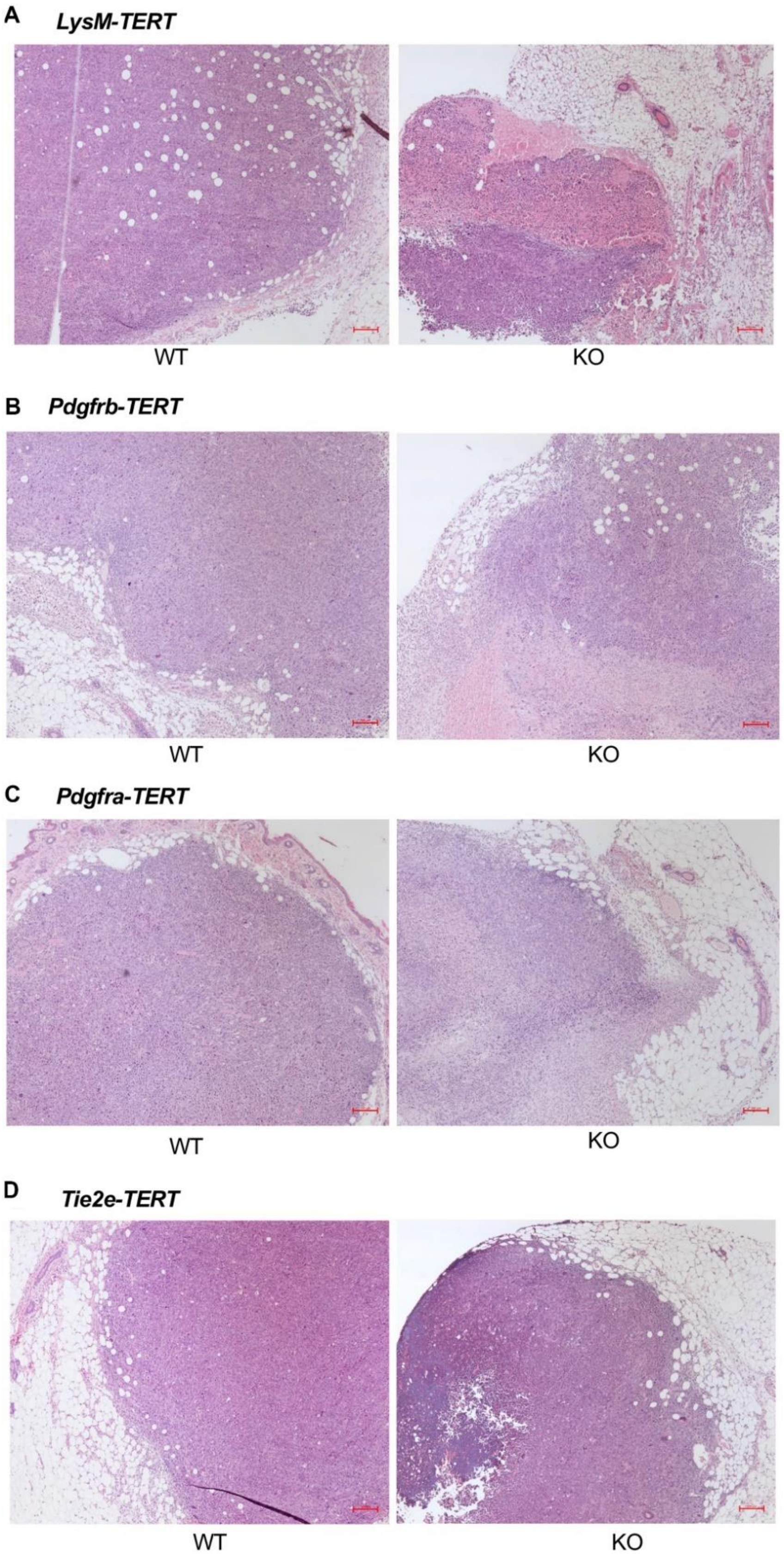

Supplementary Figure 2. E0771 tumor sections from indicated models stained with H&E. (**A**) LysM-TERT. (**B**) Pdgfrb-TERT. (**C**) Pdgfra-TERT. (**D**) Tie2e-TERT.

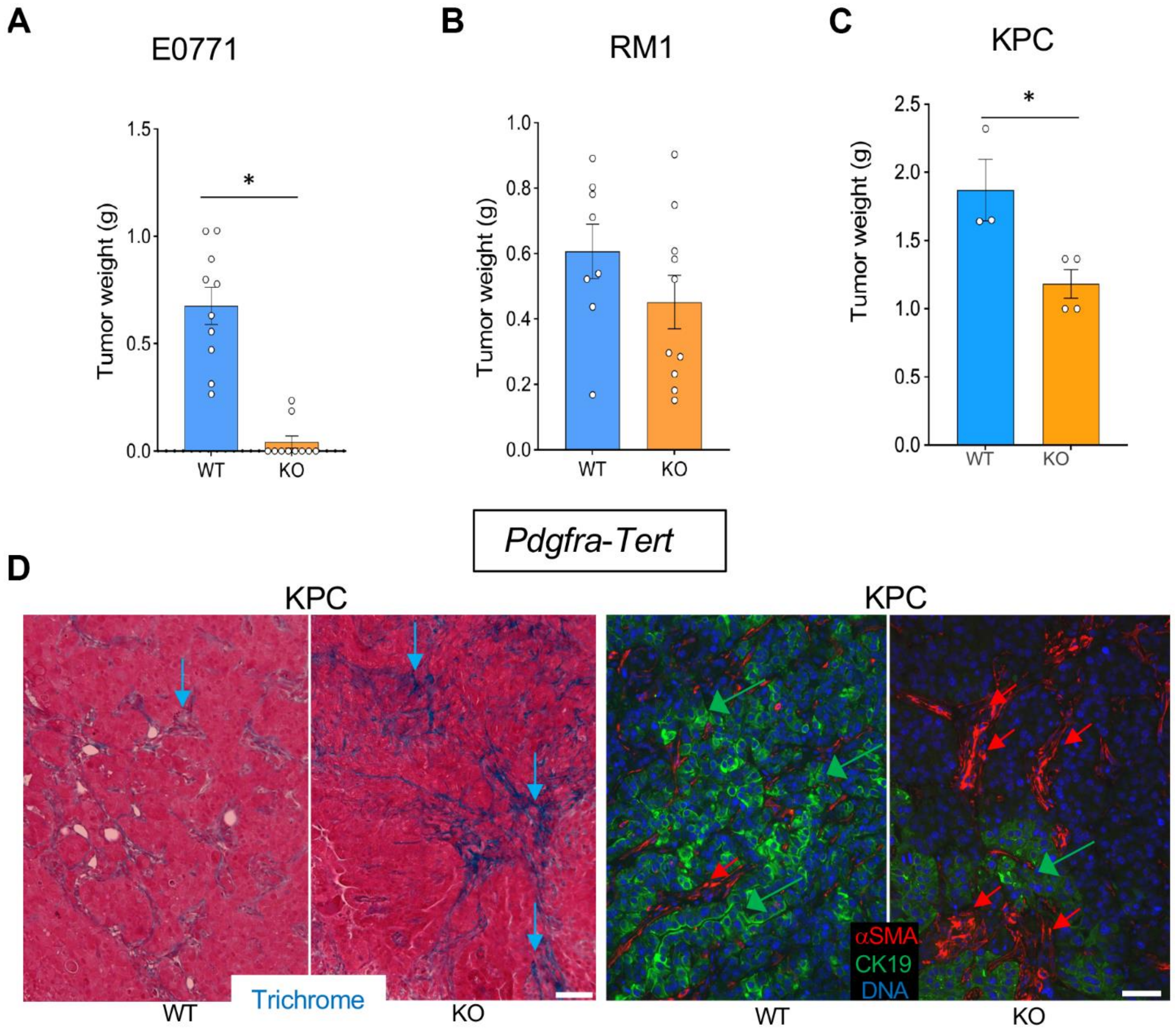

Supplementary Figure 3. Tert KO in mesenchymal cells suppresses growth of pancreatic tumors. Tissues resected from Pdgfra-Cre; $TERT^{fl/fl}$ (KO) and Pdgfra-Cre; $TERT^{fl/+}$ (WT) littermates were analyzed once one of the groups developed critical tumor burden. (**A**) E0771 tumor weight upon resection. (**B**) RM1 tumor weight upon resection. (**C**) KPC tumor weight upon resection. (**D**) Representative paraffin sections of KPC tumors stained with Trichrome (left) or subjected to CK19 and α-SMA IF (right) revealing epithelial cells (green arrows) and myofibroblasts (red arrows). Scale bar: 100 µm. Shown are mean +/- SEM, *P<0.05, Student's t-test.

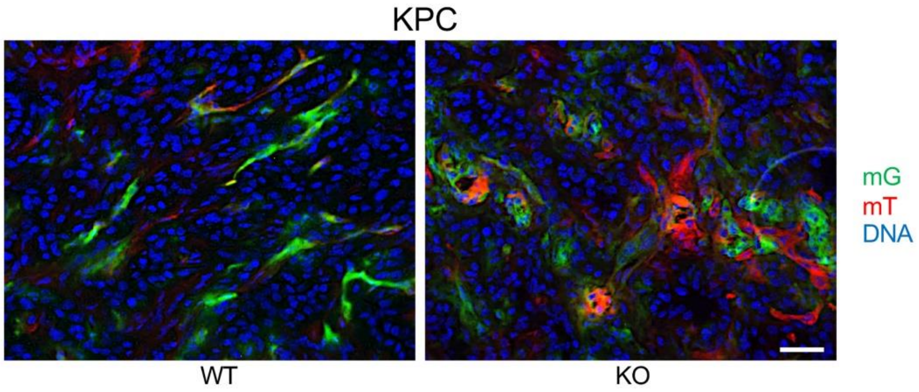

Supplementary Figure 4. Tert KO in EC results in vasculature dysfunction. Paraffin sections of representative KPC tumors from Tie2e-Cre; mTmG; TERT$^{fl/fl}$ (KO) and Tie2e-Cre; mTmG; TERT$^{fl/+}$ (WT) littermates were subjected to GFP and RFP IF. Scale bar: DNA is blue. 100 μm.
